# Automated Quantitative Analysis of *ex vivo* Blood-Brain Barrier Permeability Using Intellesis Machine-Learning

**DOI:** 10.3389/fnins.2021.617221

**Published:** 2021-04-16

**Authors:** Michael Nesbit, John C. Mamo, Maimuna Majimbi, Virginie Lam, Ryusuke Takechi

**Affiliations:** ^1^Faculty of Health Sciences, Curtin Health Innovation Research Institute, Curtin University, Perth, WA, Australia; ^2^School of Population Health, Faculty of Health Sciences, Curtin University, Perth, WA, Australia

**Keywords:** blood-brain barrier, IgG extravasation, machine-learning, immunofluorescence, laminin-α4, quantitation, Intellisis, segmentation

## Abstract

**Background:**

An increase in blood brain barrier permeability commonly precedes neuro-inflammation and cognitive impairment in models of dementia. Common methods to estimate capillary permeability have potential confounders, or require laborious and subjective semi-manual analysis.

**New method:**

Here we used snap frozen mouse and rat brain sections that were double-immunofluorescent labeled for immunoglobulin G (IgG; plasma protein) and laminin-α4 (capillary basement membrane). A Machine Learning Image Analysis program (Zeiss ZEN Intellisis) was trained to recognize and segment laminin-α4 to equivocally identify blood vessels in large sets of images. An IgG subclass based on a threshold intensity was segmented and quantitated only in extravascular regions. The residual parenchymal IgG fluorescence is indicative of blood-to-brain extravasation of IgG and was accurately quantitated.

**Results:**

Automated machine-learning and threshold based segmentation of only parenchymal IgG extravasation accentuates otherwise indistinct capillary permeability, particularly frequent in minor BBB leakage. Comparison with Existing Methods: Large datasets can be processed and analyzed quickly and robustly to provide an overview of vascular permeability throughout the brain. All human bias or ambiguity involved in classifying and measuring leakage is removed.

**Conclusion:**

Here we describe a fast and precise method of visualizing and quantitating BBB permeability in mouse and rat brain tissue, while avoiding the confounding influence of unphysiological conditions such as perfusion and eliminating any human related bias from analysis.

## Introduction

Alterations in cerebral capillary blood-brain barrier (BBB) are increasingly recognized as central in maintaining optimal brain and cognitive functioning (Zlokovic, 2008). A number of studies report that disruption of BBB is one of the earliest signs of neurodegenerative disorders such as Alzheimer’s disease and in multiple sclerosis (Nation et al., 2019). Consistently in animal model studies of neurodegeneration, BBB dysfunction resulted in significant blood-to-brain extravasation of plasma-derived proteins, leading to elevated neuroinflammation, events that preceded neurodegeneration and cognitive decline (Elahy et al., 2015; Takechi et al., 2017). However, BBB disruptions observed in neurodegenerative disorders are often subtle, resulting in a modest amount of protein leakage (Takechi et al., 2010), as opposed to more severe cerebrovascular damages seen in stroke and traumatic brain injury causing microhemorrhage (Mao et al., 2018; O’Keeffe et al., 2020).

Commonly utilized methods to determine permeability of the BBB in animal models include the *ex vivo* measurement of cerebral extravasation of exogenous dyes such as fluorescein and Evans Blue that are injected intravenously. Potential confounders include dosing; differential binding of dyes between treatment groups; metabolic effects, including on the vascular endothelium. Another major confounder of exogenous dye tracing is the requirement to *in vivo* perfusion wash. Acknowledging the importance to remove blood from the vasculature to measure the cerebral extravasation of dye, perfusion of animals introduces another layer of unphysiological conditions, which may confound the detection of subtle BBB alterations. These include the use of non-physiological perfusing solution (e.g., chilled PBS and fixative), artificial intravascular flow and pressure, and prolonged application of anesthesia (Thal et al., 2012). Moreover, tissue collected as described for measures of capillary permeability may be unsuitable for other measures of interest, requiring more mice to meet study objectives (Saunders et al., 2015).

The immunodetection of cerebral extravasation of endogenous proteins such as immunoglobulin G (IgG) and albumin may serve as preferential surrogate markers of BBB-permeability, however require either pre-perfusion to remove intravascular fluorescence, or tedious identification and demarcation of intravascular vs. parenchymal fluorescence of protein. To overcome these limitations, here we describe a novel automated method with machine-learning to achieve highly sensitive detection of cerebral extravasation of endogenous IgG, not requiring perfusion of animals prior to tissue collection.

## Materials and Methods

### Method Overview

**FIGURE 1:**
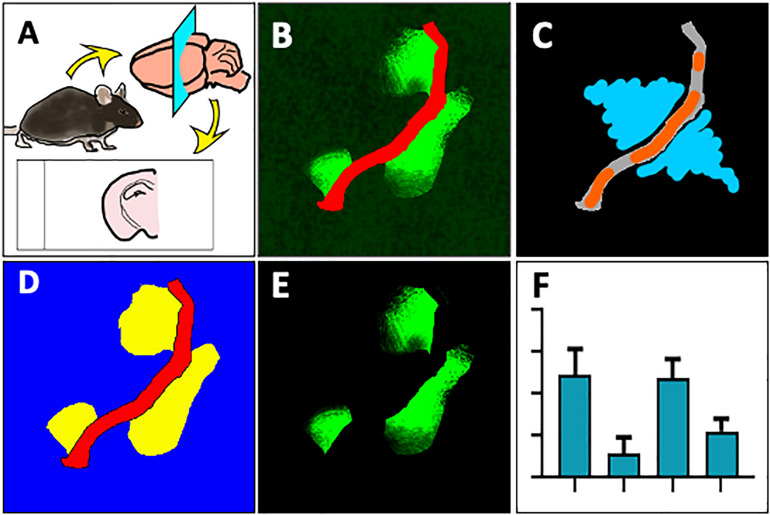
Overview of method to automatically measure IgG extravasation within ZEN software. **(A)** Immediately after culling by cervical dislocation, brain tissue was collected and snap frozen in liquid nitrogen. 20 μm sections were cut onto slides, then fixed in 4% paraformaldehyde. **(B)** Sections were immunostained against mouse immunoglobulin G (green) and laminin-α4 (red) and imaged on a spinning disc confocal microscope. **(C)** 3-D images were flattened to maximum intensity projections and a ZEN Intellisis model was trained on several random images of varying staining intensity by labeling blood vessel morphology (orange) and background (blue). **(D)** An Image Analysis setting was designed with three automatically segmented classes: Laminin-α4 (red), parenchyma (blue), and parenchymal IgG (yellow). **(E)** Parenchymal IgG pixel intensity was quantified. **(F)** The Image Analysis setting was applied to multiple images to automatically produce a data table for downstream analysis.

Snap frozen brain hemispheres were sectioned using a cryotome and fixed with paraformaldehyde (Figure 1A), followed by immunofluorescent double labeling with antibodies against mouse or rat IgG and laminin-α4. Samples were then imaged on a confocal microscope and maximum intensity projection images were converted for use in Zeiss ZEN Blue software (Figure 1B). Blood vessels and parenchymal regions were segmented by training a Machine Learning Intellesis module, and IgG signal was identified using a pixel intensity threshold in the parenchymal segment (Figure 1D). Pixel intensity of the IgG channel within the IgG subclass was measured (Figure 1E). Automatic generation of result data tables based on various features, and batch image analysis can be achieved using this new method (Figure 1F).

### Materials

Materials required for immunofluorescent slide staining and image capturing are indicated in their respective sections. Quantitation was performed on ZEN Blue 3.1 software (Carl Zeiss Microscopy GmbH) requiring modules: ThirdPartyImport, Intellesis, and ImageAnalysis. Minimum system requirements: Intel^®^ Core^TM^ i5-4670 4- Core 3.0 GHz, 8 gb RAM, 250 gb free hard disk space.

### Animals

Male 6-week old C57BL6/J mice and female PVG rats were purchased from Animal Resources Centre (WA, Australia). The animals had *ad libitum* access to the standard rodent chow and water. We have used previously established models of mild-severe BBB disruption in our laboratory for the validation of the new method developed (Lavender et al., 2020). The animal procedure described was approved by Curtin Animal Ethics Committee (Project Approval AEC2016-25 and ARE2019-13).

### Tissue Collection and Processing

Animals were anesthetized with 4% gaseous isofluorane and exsanguinated via cardiac puncture followed by cervical dislocation. The left hemisphere of brain tissue was immediately removed and frozen in liquid nitrogen, then stored at –80°C. Brain tissue was sliced into 20 μm coronal sections using a Leica CM1520 cryostat onto poly-L-lysine coated slides (Trajan P7511) and either immediately frozen or immediately fixed in 4% paraformaldehyde (Sigma P6148) for 10 min on ice.

### IgG and Laminin-α4 Staining Protocol

Samples were washed 3× in 0.1 M phosphate buffered saline (PBS) pH 7.4. Fixed brain sections were incubated overnight at 4°C in 1:200 goat anti-laminin-α4 (R&D systems, AF3837) in Antibody Signal Enhancement Buffer (PBS with 10 mM glycine, 0.05% Tween20, 0.1% Triton, 0.1% BSA) as per the protocol described by Rosas-Arellano et al. (2016).

Slides were then washed 3× in PBS and incubated with donkey anti-goat Alexa555 (Abcam ab150130) 1:500 for 2 h at room temperature and washed 3× in PBS, followed by a further 1 h incubation with anti-mouse Alexa488 (Thermo Fisher A-11029) 1:100 or anti-rat Alexa 488 (Thermo Fisher A-11006) in PBS. Slides were washed 1× in PBS, then nuclear counterstained with DAPI. Slides were washed 3× in PBS, then mounted in an anti-fade mounting medium.

### Image Capture

Stained sections were imaged using an UltraVIEW Vox Spinning Disk Confocal Microscope (PerkinElmer) with a Hamamatsu C9100-50 EMCCD coupled with Volocity image acquisition software. A minimum of five 3-dimensional images consisting of 21 x 2D z-stacks from each mouse or rat (20× objective, N.A. 0.95, 1,000 × 1,000 pixels, 346 μm × 346 μm, 1 μm Z-step size) were captured at random positions throughout the hippocampus and cerebral cortex. To address the Intellisis module’s incapability to perform segmentation on 3D images, Maximum Intensity Projections were produced using ImageJ’s “Z-project” processing function. This also significantly reduced data storage and processing burden. XY points for this purpose were designated the “time” parameter, enabling quantitation of multiple images within one TIFF file. Images stored in the Zeiss native.czi format do not require conversions described here.

### Intellisis Segmentation of Blood Vessels and Extravascular IgG Quantitation

Pre-requisites:

• The following modules within ZEN 3.1 or above are licensed: ImageAnalysis, Intellesis, Third Party Import.
• Images are in a.czi file format. For images acquired on other platforms, import into FIJI/ImageJ using the BioFormats Import plugin and save as TIFFs. TIFFs can be imported into ZEN using the option File > Import/Export > BioFormats Import.
• Multiple Z-stack images can be flattened using the “Z Project” tool in FIJI/ImageJ or the “Make Maximum Intensity Projection” tool in the orthogonal view in ZEN.
• A “training set” of about 10 images has been derived from the experimental dataset.

1. Train an Intellesis Model on the laminin- α4 single channel image

a. Create new Intellesis Trainable Segmentation model and click “Start Training.”
b. Select Segmentation: “Basic Features 33” and Postprocessing: “Conditional Random Field.”
c. Click “Import Images” to open the single channel laminin a4 training set images. Choose “Training Mode” as “Single Channel,” and select the Laminin-a4 channel.
d. Note: Imported sample images must have pixel and channel match to the experimental images.
e. Under the “Labeling Options” tab, use the paint brush, eraser and brush width tools to label some input pixels as “Object” for Laminin- α4 or “Background.”
f. Note: It is not required to label the whole object. Carefully label boundaries between vessels and background, focusing especially on weaker signal.
g. Click “Train and Segment” to create a “Segmentation” channel. Check where the software has made mistakes and then re-label misclassified regions. Check the “Confidence” channel—label the regions of low confidence indicated by dark grey pixels.
h. Repeat steps 1d. to 1e. on the training set until the Intellesis model classifies accurately. Click “Finish.”

2. Determine an IgG signal threshold intensity using the “Profile” tab on the left side of the image viewer.

For example, if background values never exceed 2,000 and IgG signal is around 5,000, choose a threshold of 3,500.

3. Create a new Image Analysis Setting to automatically quantitate IgG.

a. In the “Image Analysis” tab click “Setup Image Analysis.”
b. Step 1: Add and name the following classes:
  i. Class 1: Laminin
  ii. Class 2: Extravascular segment
  iii. Class 2 Subclass: Extravascular IgG

Note: Ensure the correct target Channels for each class are selected.

c. Step 2: Leave “Frame” as “Interactive.”
d. Step 3: Automatic Segmentation:
  i. Click the Laminin class and change “Segment by Global Thresholding” to “Intellesis Trainable Class Segmenter” and select Model as created in Step 1.
  ii. Select “Model Class” as “Object.” Select “Fill holes.”
  iii. Click the Extravascular Class and repeat Step 3di.
  iv. Select “Model Class” as “Background.” Do not select “Fill Holes.”
  v. Select “Binary,” choose “Erode” and set “Count” as 1 to make a 1 pixel buffer around blood vessels.
  vi. Click the IgG class and set the “Threshold” to the value determined in Step 2 with 0% tolerance.
  vii. Select “Smoothing,” choose “Lowpass” and set “Size” as 3 to reduce IgG signal noise.
e. Step 4: Region Filter:
  i. It is not necessary to filter segmented objects, but it may be required to set a minimum area of approximately 5 μm^2^ to exclude unspecific signal.
f. Step 5: Leave “Interactive Segmentation” as “Interactive.”
g. Step 6: Features:
  i. To record vascular density, click the Sum Laminin class (Σ) and select “Edit.”
  ii. Add features “Image Name,” “Area,” “Area Percentage,” and “Area Frame.”
  iii. Click the Sum IgG class (Σ) and select “Edit.”
  iv. Add features “Intensity Sum of Channel X” and “Intensity Pixel Count of Channel X.”
  v. For all features, select “Copy to Sum Laminin class (Σ)” to consolidate all data into one table.
h. Step 7: Results Preview.
  i. Check segmentation and results table, unchecking “Show Objects” in the Analysis tab to compare with the raw image.
  ii. Click Finish

4. Open experimental datasets and click “Analyze” in the “Analysis” tab to run the Image Analysis Program.
5. To extract the data table, select “Sum Laminin class (Σ)” at the bottom “Analysis” Tab and click “Create Table.” This table can be copied into Microsoft Excel for collation and analysis.
6. Check through images to confirm accurate segmentation and exclude unwanted data. Alternatively, click “Analyze Interactively” to adjust the analysis frame (step 2) or manually correct segmentation (step 5).
7. Repeat steps 2–6 with the remaining experimental data set.

Note: Batch Analysis can be done on multiple.czi files through the Processing tab > Batch > Batch Method > Image Analysis Program. Macros are also available to automatically construct data tables from batch analyses.

All analysis was performed on a PC workstation (Intel Core (TM) i7-4770 CPU, 24 GB RAM, Windows 7 × 64).

## Results and Discussion

### Laminin-α4 and IgG Double Immunofluorescence Accentuates the Detection of Mild Increases in BBB Permeability

The assessment of subtle changes in cerebrovascular BBB permeability is increasing in importance in neuroscience research. The detection of such delicate breaches of the BBB remains elusive and may be confounded by various factors including the cellular effects of exogenous dye and extended use of anesthesia. Moreover, historical approaches to determine BBB permeability are laborious due to manual selection of extravasation and analysis is often subjected to human bias. Here, we present a new methodology of highly sensitive automated detection of BBB disruption in mouse and rat brain that may mitigate the aforementioned major confounding issues.

We utilized laminin-α4 staining to precisely identify the microvascular network. The laminin-α4 subunit comprises laminin isoform 8 (new nomenclature laminin-411) and is a major subendothelial basal membrane component of animal CNS vasculature, including capillaries (Frieser et al., 1997; Iivanainen et al., 1997). This is in contrast to astrocytic laminin-α2 which comprises laminin-211, deposited around larger cerebral blood vessels by astrocytic end-feet (Jucker et al., 1996). The laminin-α4 staining produced a distinct clear boundary between the intraluminal and parenchymal environments. No background or non-vessel staining was observed. We also confirmed that laminin-α4 stained all visible blood vessels within the field of view, which was identified by using DAPI nuclear counterstain. By training using laminin-α4 stained blood vessel images, Intellesis was able to precisely learn the morphological patterns of the microvasculature (Figure 1C) and discriminate the vessel area vs. non-vessel area (Figure 1D). Concurrently, an IgG subclass within the parenchymal segment was identified using an intensity threshold (Figure 1E), and the underlying IgG pixel intensity and area was quantitated (Figure 1F).

**FIGURE 2:**
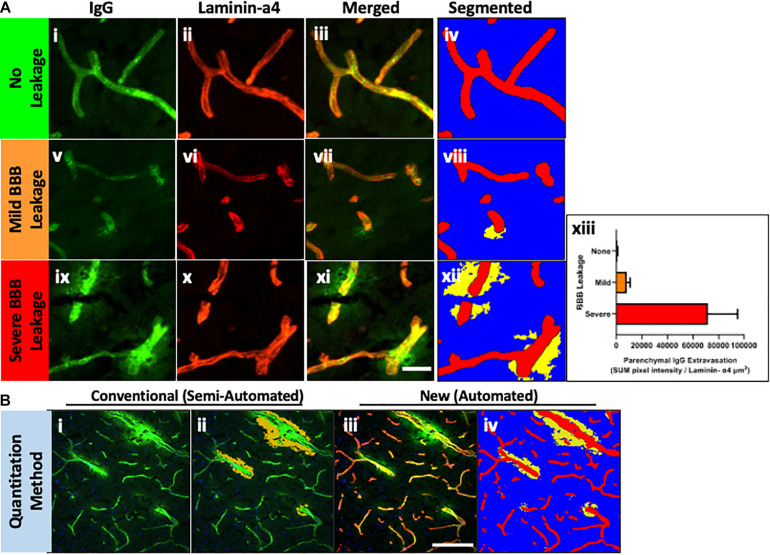
**(A)** Blood vessel staining using laminin-α4 enhances contrast and detection of mild BBB leakage events. Maximum intensity projections of 20 μm mouse brain sections, stained using anti-mouse IgG Alexa488 (i,v,xi) (green), anti-laminin-α4 (i,vi,x) (red), and merged (iii,viii,xii). Images were automatically segmented using the laminin-α4 Intellesis model to reveal only parenchymal IgG staining above a threshold intensity, accentuating differences in BBB leakage: none (iv), mild (viii) and severe (xii). (xiii) Automatic quantitative data of representative images using ZEN ImageAnalysis. Scale bar 20 μm. **(B)** Comparison between methods for quantitation of parenchymal IgG extravasation. Single-channel anti-mouse Alexa488 images (i) are traditionally semi-quantitatively assessed (ii) to identify BBB leakage using a Magic Wand tool (orange), which automatically selects neighboring pixels of similar intensity to those manually selected. User input is required to manually erase intravascular IgG selected by the Magic Wand tool. The method described in this paper uses Machine Learning to identify blood vessels (red) and quantitate parenchymal IgG staining (iii) based on a pre-determined IgG intensity threshold (iv) (yellow). Scale bar 100 μm.

Thereafter, the ZEN Image Analysis protocol was applied to example images without IgG extravasation, mild leakage and severe leakage, to test whether the method can quantitatively analyze parenchymal IgG extravasation of varying degrees. As indicated in Figures 2Ai–iv, Intellesis segmentation was able to precisely identify the blood vessel area in the images with no IgG leakage, and intensity thresholding within the parenchymal segment resulted in no quantitative detection of IgG voxel intensity (Figure 2Axiii). In the images with mild IgG leakage, Intellesis was also able to identify the blood vessels precisely (Figures 2Av–viii), and the area of parenchymal IgG extravasation was evident (Figure 2Aviii), which allowed accurate automated quantitation of intensity within this area (Figure 2Axiii). Similarly, in the example images of severe BBB disruption (Figures 2Aix–xi), the segmentation of the blood vessels by Intellesis was successful, enabling the quantitation of the parenchymal IgG extravasation (Figure 2Axiii).

In biological specimen samples derived from preclinical models of neurodegenerative disorders, mild BBB leakage events are much more numerous than severe BBB leakage events, however, are visually less obvious. The marked boundary between the intraluminal and parenchymal environments of small capillaries defined by laminin-α4 masking, removes all ambiguity of IgG staining location which previously concerned investigators, and significantly improves the detection of subtle leakage patterns (Figure 2viii).

### Improvements in Accuracy and Time Burden Compared to a Conventional Method

**FIGURE 3:**
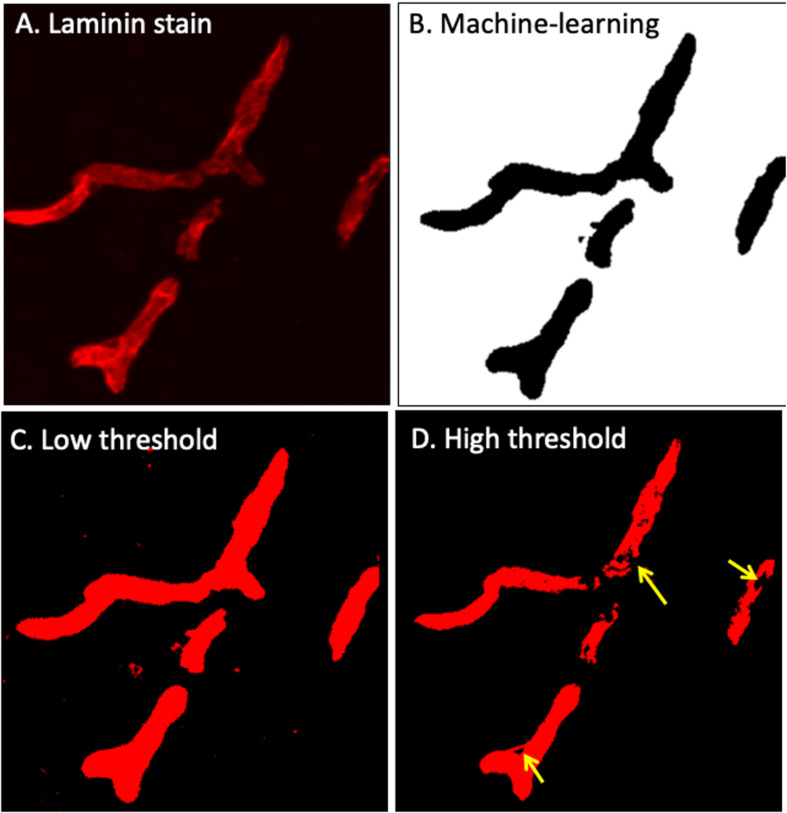
Laminin-α4 staining provides a clear indication of the BBB **(A)**, however pixel intensity varies considerably. Intellesis is able to recognise high and low intensity pixels accurately **(B)**, unlike classical intensity thresholds which result in either too much **(C)** or too little **(D)** capillary segmentation.

We compared the new IgG quantitation method established in this paper to our conventional “manual” IgG quantitation protocol. Single-channel anti-mouse 488 images (Figures 2Bi, 3A) are traditionally semi-quantitatively assessed to identify BBB leakage. As described previously (Takechi et al., 2013), Volocity (Figure 2Bii PerkinElmer) software is used to manually select image regions of vascular plasma protein leakage by a blinded investigator (Figure 3B). The manual method can result in over-selection of background pixels, unintentional measurement of intravascular IgG, or subtle leakage events are undetected. Using the image example in Figure 2B, the conventional method identified an average pixel intensity of 3,495 within the selected measurement region, which is very close to background intensity as determined using a “Line Profile.” Impressively, the new method described in this paper identified pixels of an average intensity of 6,012, indicating a significant improvement in recognition accuracy of true positive IgG staining.

Careful manual quantitation of large datasets can occupy a trained investigator for several weeks. The high-throughput method described in this paper allows researchers to identify modest IgG extravasation en masse with minimal user input (Figure 3). Utilizing this protocol, thousands of images can easily be automatically quantitated, which also removes the issue of user bias. This protocol markedly reduces the time required to manually quantitate large image sets, allowing more time for data acquisition or obtaining larger numbers of samples to enhance statistical power.

### Data Reproducibility

The reproducibility of the data is presented in Table 1. The segmentation/quantitation method was applied to 14 randomly selected images, and the process was repeated three times. The CV percentage varied from 2.5 to 11.4%, indicating the high reproducibility of the data by using this novel automated method.

**TABLE 1:** Data reproducibility.

Image no.	IgG extravasation (pixel intensity)			CV (%)
	
	Trial 1	Trial 2	Trial 3	
1	32,891,194	29,474,127	32,067,765	5.67
2	674,000,000	622,000,000	692,000,000	5.49
3	22,758,496	19,888,229	21,030,290	6.81
4	195,000,000	186,000,000	193,000,000	2.47
5	46,678,740	42,382,137	46,144,489	5.20
6	31,895,594	28,146,579	31,676,728	6.88
7	53,381,611	47,422,500	50,657,045	5.91
8	174,000,000	166,000,000	171,000,000	2.37
9	54,749,446	48,150,383	54,016,096	6.91
10	52,851,403	45,784,695	49,940,009	7.17
11	3.54e+008	3.37e+008	3.51e+008	2.61
12	4,492,003	3,670,690	4,518,946	11.41
13	9,893,960	9,237,617	9,827,299	3.74
14	36,122,998	30,377,113	35,652,321	9.37

### Method Applicability

The applicability of the automated selection of peri-vascular IgG extravasation in order to assess the BBB integrity was tested on several images from thalamus as presented in Supplementary Figure 1. We confirm that the developed method accurately identify the parenchymal IgG extravasation in other regions of the brain including hypothalamus.

## Conclusion

In conclusion, this machine-learning method produces a high-signal to noise ratio to measure BBB impairment and indeed, advantageous to perfusion of exogenous dyes such as Evans Blue, without altering physiological conditions which may affect BBB permeability. Laminin-α4 co-staining with IgG drastically accentuates the determination of subtle changes in BBB permeability which are otherwise undetectable, while facilitating expeditious and intelligent automated quantitative analysis. This method is significantly superior to other existing methods because it eliminates any potential bias by the investigator(s), avoids labourious image analyses/processing by the investigator(s), and is highly reliable and reproducible.

## Data Availability Statement

The original contributions presented in the study are included in the article/Supplementary Material, further inquiries can be directed to the corresponding author/s.

## Ethics Statement

The animal study was reviewed and approved by the Curtin Animal Ethics Committee.

## Author Contributions

MN was involved in the conceptualization, data curation, formal analysis, and investigation and writing manuscript. JM contributed to the funding acquisition and resources and manuscript writing. MM validated the methodology. VL contributed to the funding acquisition, animal experiments and sample collection, and method development. RT developed the study concept, obtained the funding, supervised the project, and wrote the manuscript. All authors contributed to the article and approved the submitted version.

## Conflict of Interest

The authors declare that the research was conducted in the absence of any commercial or financial relationships that could be construed as a potential conflict of interest.
