# Supplementary material for: Automated Quantitative Analysis of ex vivo Blood-Brain Barrier Permeability Using Intellesis Machine-Learning
Source: Front Neurosci. 2021 Apr 16;15:617221. doi: 10.3389/fnins.2021.617221 (PMC8086794; doi:10.3389/fnins.2021.617221)
Supplement: Supplementary file 1 [file Image_1.pdf]

Supplementary Material

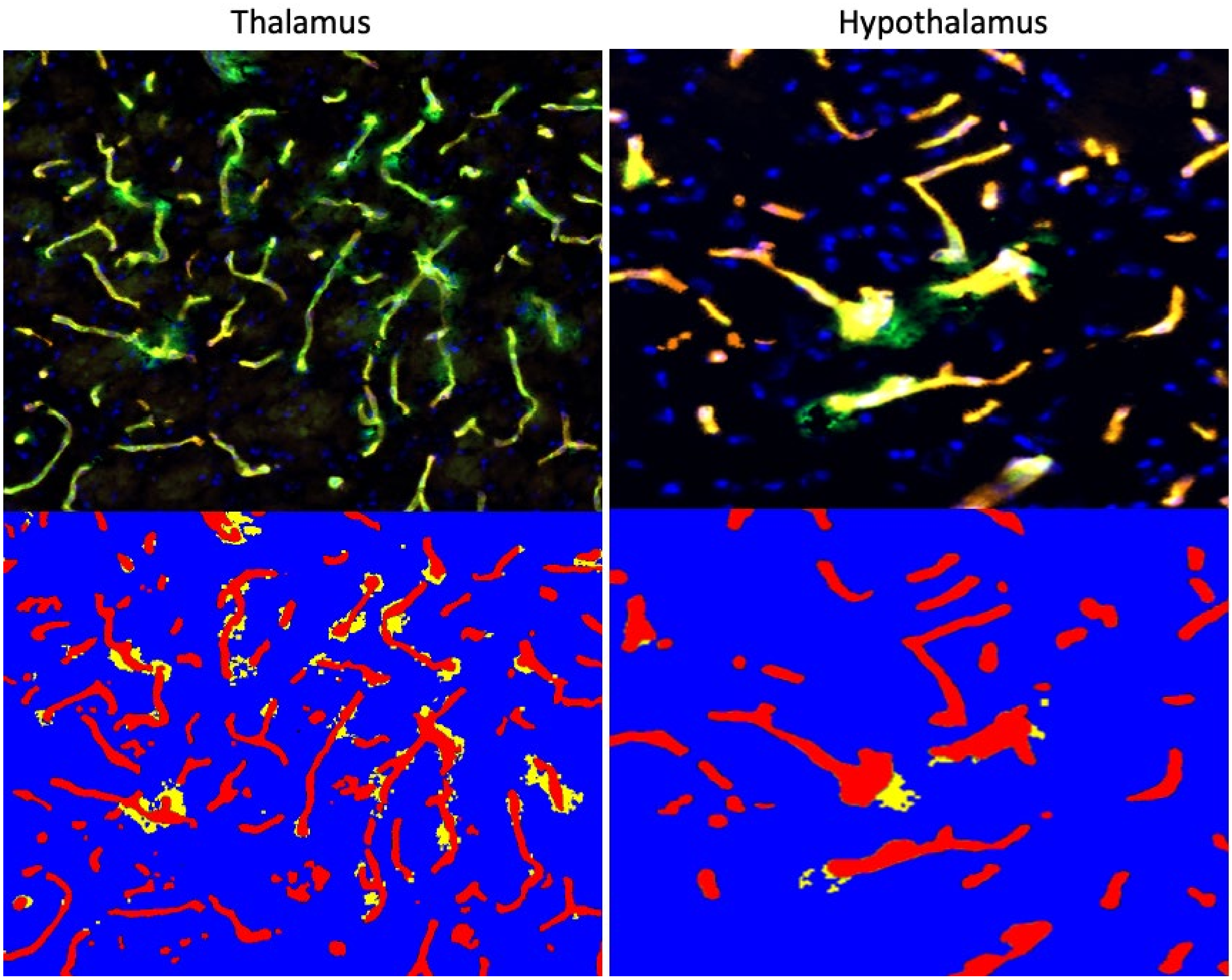

Supplementary Figure S1. The Intellesis machine-learning automated selection of peri-vascular IgG extravasation was applied to the images captured from thalamus and hypothalamus regions. Representative images show accurate selection of parenchymal IgG leakage, confirming the applicability of the method to other brain regions.
